# Haemodynamic changes during the peri-extubation period using bioreactance flow monitoring

**DOI:** 10.1186/cc10831

**Published:** 2012-03-20

**Authors:** J Thirsk, D Magimairaj, A Douiri, D Hadfield, P Hopkins

**Affiliations:** 1 King's Health Partners, London, UK

## Introduction

Here we present a prospective, observational study examining the effect of extubation on cardiac index, measured by bioreactance (Nicom Cheetah), in critically ill patients with or without a history of left ventricular impairment [1]. A number of simple interventions are known to improve the process of weaning patients from mechanical ventilation. Despite this progress, the pathophysiology underlying failure to wean remains incompletely understood. In particular, the role of cardiac ventricular dysfunction may be underestimated [2].

## Methods

**Figure 1:**
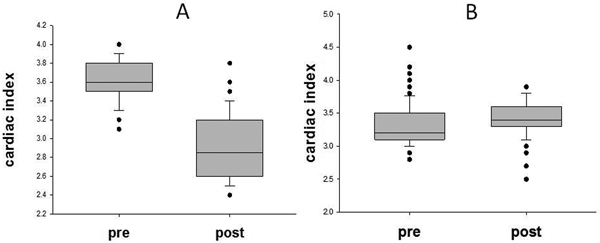


Cardiac index was measured by bioreactance monitoring at 30-second to 60-second intervals for 1 hour pre and 1 hour post extubation. Individual data were presented by box plot, showing median and interquartile ranges (Figure 1). Combined results from multiple patients in each test group were analysed by covariance (Stata version 11.2).

## Results

Group A (*n *= 5) had impaired left ventricular systolic function, documented on formal transthoracic echo, of which three had ejection fractions <25%. One patient in this group failed extubation due to cardiogenic pulmonary oedema. Group B (*n *= 6) had normal systolic function. Figure 1 shows representative absolute data obtained from a patient in each group. There was a statistical difference between the two groups (*P *= 0.02). In the impaired LV group, the cardiac index fell from 3.2 l/minute/m^2 ^(± 0.5) to 2.9 l/minute/m^2 ^(± 2.5).

## Conclusion

In this small observational study we demonstrated a consistent fall in cardiac index post extubation in patients with known cardiac ventricular dysfunction when compared with patients with normal hearts. These data suggest that bioreactance monitoring may be valuable during spontaneous breathing trials and extubation.

## References

[B1] Benomar B Ouattara A Estagnasie P Brusset A Squara P Fluid responsiveness predicted by non-invasive bioreactance-based passive leg raise test Intensive care Med 2010 36 1875 1881 10.1007/s00134-010-1990-6 20665001

[B2] Papanikolaou J Makris D Saranteas T New insights into weaning from mechanical ventilation: left ventricular diastolic dysfunction is a key player Intensive Care Med 2011 37 1976 1985 10.1007/s00134-011-2368-0 21976188

